# From Neonatal Hearing Screening to Intervention: Results of the Dutch Program for Neonatal Hearing Screening in Well Babies

**DOI:** 10.3390/ijns4030027

**Published:** 2018-08-01

**Authors:** Noëlle Uilenburg, Catharina Van der Ploeg, Rosanne van der Zee, Anneke Meuwese-Jongejeugd, Bert van Zanten

**Affiliations:** 1 Dutch Foundation for the Deaf and Hard of Hearing Child, Lutmastraat 167, 1073 GX Amsterdam, The Netherlands; 2 TNO Department of Child Health, Schipholweg 77, 2316 ZL Leiden, The Netherlands; 3 Center for Population Screening, The Dutch Institute for Public Health and the Environment, Antonie van Leeuwenhoeklaan 9, 3721 MA Bilthoven, The Netherlands; 4 ENT Department, University Medical Center Utrecht, Heidelberglaan 100, 3584 CX Utrecht, The Netherlands

**Keywords:** neonatal hearing screening, population screening program, hearing loss, young children, early intervention

## Abstract

In the Netherlands, Youth Health Care services (YHC) have been carrying out neonatal hearing screening (NHS) in newborns since 2006. The aim of the NHS is to identify children with permanent hearing loss, so that intervention can be started before the age of 4 months. Early detection of hearing loss is important, as children who start intervention early have been shown to develop better. This article describes the structure and performance of the NHS carried out by the YHC, the quality of the program, and the timeliness of the start of intervention. Since its implementation, the NHS has been audited annually in order to monitor the program’s quality. Monitoring reports and data from the Dutch Foundation for the Deaf and Hard of Hearing Child were used in this study. For many years, results have shown the NHS to be a stable screening program of high quality. The participation rate is high, refer percentage low, and the timeliness of the program is continually improving. Although the timeliness of post screening diagnostics and intervention need most improvement as they do not always meet the target times, this has improved over recent years.

## 1. Introduction

In the Netherlands, the Youth Health Care services (YHC) have been performing neonatal hearing screening (NHS) in newborns since 2006 [1,2]. NHS has been carried out in children in neonatal intensive care units (NICU) in hospitals since 2002. The aim of NHS is to trace children with permanent hearing loss of a minimum of 40 dB in one or both ears, so that an appropriate intervention may be given before the age of 4 months. Due to the strong Netherlands tradition of giving birth at home or in outpatient services, NHS is also mainly performed at home. Hearing screening is part of the standard package provided by the YHC, and is financed by the local authorities. The NHS replaced the original Ewing/CAPAS screening tests, which used to be carried out at around the age of 9 months. These methods were substituted after mounting scientific evidence began to show that children in whom an intervention for hearing loss was started before the age of 6 months were shown to develop better in various ways, such as by having larger active and passive vocabularies, being able to articulate more clearly, having better auditory function, reaching higher reading levels, having better parent-child interaction, and experiencing fewer socio-emotional problems [3,4,5].

In addition, the development of new screening apparatus has enabled newborns to be screened at home, meaning that identification methods of children with hearing loss have been undergoing a great change internationally. Most importantly, not only has detection itself been fundamentally changed by the introduction of NHS, but also hearing diagnostics, hearing rehabilitation, and the early intervention of children with hearing loss—and if we also include the rise and effect of early cochlear implantations, we can see that there has been an even more radical change. Moreover, the increased accessibility of early interventions for children with moderate hearing loss (40–60 dB) and developments in the quality of hearing aids have also contributed to the series of changes that have considerably increased the developmental opportunities of children with hearing loss.

However, these changes have not evolved on their own—a lot of hard work has gone into accomplishing them. Possibly the most important factor in this process has been that since the introduction of NHS, the focus has consistently been on maintaining and improving the quality of the screening program, diagnostics, and intervention. The Dutch Institute for Public Health and the Environment’s Center for Population Screening (RIVM-CvB), directs the NHS on behalf of the Dutch Ministry of Health, Welfare and Sports. The underlying principle is that every population screening program should satisfy the public values of quality, accessibility, and affordability. To this effect, national guidelines, the organization of delivery, and quality requirements are laid down in a procedure manual [6]. In turn, the RIVM-CvB is advised on carrying out its management remit by a program committee.

A set of quality indicators has been created for the NHS, and each year the program is evaluated on behalf of RIVM-CvB. Since its implementation, this monitoring procedure has been carried out by The Netherlands Organization for Applied Scientific Research (TNO) [7]. The main quality indicators are: (a) participation, (b) timeliness, and (c) refer percentages, or percentages referred to speech and hearing centers (SHC). The SHCs and institutions for the care of children with sensory handicaps (early intervention centers) also maintain and evaluate quality registers, and, if necessary, implement improvement trajectories.

This article describes the structure and performance of the NHS delivered by YHC services, the quality of the program, and the timeliness of the start of intervention. The hearing screening carried out in NICUs falls outside the scope of this article as this is a standard part of NICU treatment delivered by the hospital.

## 2. Materials and Methods

### 2.1. Screening Protocol

Two screening methods are used in the Netherlands: the otoacoustic emission (OAE) method, and the automated auditory brainstem response (A-ABR) method. Both methods have a high level of sensitivity and specificity.

The OAE method measures sounds (emissions) that are produced by the outer hair cells in a well-functioning inner ear, during, and for a short time after, the processing of a sound [8]. These emissions are measured by means of a small microphone in the ear. The presence of otoacoustic emissions indicates that the hearing is functioning well up to and including the outer hair cells in the inner ear. In the Dutch program, transient evoked OAEs (TEOAEs) are elicited with brief (transient) sounds, presented at an intensity level of 80 dB SPL. TEOAEs are recorded over the frequency range of 500 to 4000 Hz. The A-ABR method measures the electrophysiological responses to sound up to the brain stem level, and these responses are compared with those of neonates with good hearing. During A-ABR screening, click stimuli are presented at 35 dB nHL, at a rate of 37 clicks per second. Each click is a composed sound that covers the frequency range of 700 to 5000 Hz.

The neonatal hearing screening test may be carried out in three stages, i.e., up to three separate screening sessions take place, as needed. Based on uniform and timely information, parents make an informed decision about their child’s participation in the program. At the first and second screening sessions, the OAE method is used. In most parts of the Netherlands, the first screening test is carried out by a member of the YHC staff at the same home visit as the heel prick test. In areas where midwives perform the heel prick test, the first screening test is done at a Well Baby Clinic at around three weeks after birth. If a satisfactory result for both ears is not obtained at the second OAE screening, then the third screening test is carried out by a regional coordinator using A-ABR. There is a 4–7 day period between each screening session. If, after the third screening, satisfactory results for both ears have not been obtained, the child is then referred to a SHC for further diagnostics. The screening procedure should be completed within 42 days of birth. Those children who are admitted to the hospital (but not to NICU) for a long period after birth, or children who have risk factors for auditory neuropathy syndrome disorders (ANSD) follow a separate NHS screening protocol. These children are screened with A-ABR screening in the first stage, because ANSD is not detected by OAE screening. This protocol is also carried out by YHC Staff.

The quality of the NHS is closely monitored by a regional coordinator. A regional coordinator also manages the OAE screeners, organizes training, and coaches the screening teams. In the monitoring process, the regional coordinator makes use of a number of functions of the Central Administration System Neonatal Hearing Screening (CANG). See data collection for more information.

### 2.2. Diagnostics

At the SHC, diagnostic tests are performed in accordance with a uniform diagnostic protocol for neonates. In this protocol, the following diagnostic tests are stipulated: auditory brainstem response measurements, impedance testing, frequency specific otoacoustic emission measurements, and behavioral/observation audiometry.

In the Netherlands there are 26 SHCs where neonatal diagnostics are carried out, which include both academic and regional centers. The aim is to have all diagnostic hearing testing completed within 92 days after birth. The results of the diagnostic tests are stored in the SHC’s electronic dossiers, and when parental permission has been obtained, also in the CANG.

### 2.3. Intervention

According to Dutch healthcare legislation, children with hearing loss of a minimum of 35–40 dB in the best ear are eligible for intervention by specialist centers for children with sensory handicaps. This is in accordance with the year 2000 position statement of the Joint Committee on Infant Hearing, which was used as a guideline for the screening program [9]. Although a more recent position paper (2007) states that all degrees of hearing loss are indicated as targeted hearing loss for NHS, with the current screening protocol these children are not able to be identified [10].

Children with unilateral hearing loss receive a follow-up at an SHC. Parents receive counselling from the SHC, and the child’s hearing loss is monitored in order to identify any deterioration over the years.

The aim was to start the intervention within 4 months for children with bilateral hearing loss, whether it be an early intervention with hearing aids via an SHC, and/or an early intervention via a specialist center for people with sensory handicaps. The Netherlands has six early intervention centers that offer a range of services, including family counselling, courses for parents (including sign language courses), group intervention, and individual intervention (hearing training, speech and language therapy, etc.). The results of the interventions were stored in (electronic) dossiers.

### 2.4. Data Collection

The results of the screening for hearing testing were obtained from a report that evaluated its national implementation (2002–2006), and also from national monitoring reports (2008–2016) [2,7]. The data that were supplied for this purpose came from the CANG (with permission), which is used by all YHC organizations to support the NHS [1,2]. After obtaining parental permission, data on diagnostic testing and intervention were obtained after NHS from the electronic dossiers of the Dutch Foundation for the Deaf and Hard of Hearing Child (NSDSK)’s early intervention center, as well as the national register of diagnostics.

## 3. Results

### 3.1. Screening and Diagnostics

Table 1 shows the participation percentage for each screening session and the percentage of children who participated in diagnostic testing after referral to an SHC. At each of the three screening sessions, participation was consistently high and always above the signal value of 98%. The aim was 100% participation in diagnostics, as there was a high risk of hearing loss in children who failed the third screening with A-ABR (35–40%). There is a number of children who go unreported each year. If it is assumed that the unreported children did participate in diagnostic tests, then the participation percentage will be considerably closer to the signal value, i.e., varying between 97.1% in 2011 and 98.7% in 2013.

Table 2 shows the referral percentages per screening session and the percentage of referrals to an SHC. At the first and second sessions, the percentages were well below the norm or signal value. Because the chance of finding a truly positive screen result, the percentage of children referred in the second stage of screening is higher than the first. The percentage of children who were referred for diagnostic testing (<0.3%) was also well under the norm of <0.5%.

Table 3 outlines the timeliness of the screening sessions and the diagnostic testing. Up to 2012, the national average often did not meet the target and signal values for timely screening. This result changed in 2013, even in the regions where screening was carried out at Well Baby Clinics. These clinics started screening at the age of approximately three weeks, which means the time remaining for follow-up screening was considerably limited. The relevant organizations have since focused on improving this, with positive results. However, the timeliness of diagnostic testing still does not yet meet the target time, i.e., where within 3 months > 95% should be completed.

The number of children with permanent bilateral hearing loss of a minimum of 40 dB detected annually, varies year by year (Table 4). In 2015, using the NHS, the YHC screened 166,312 children, 113 of whom were found to have permanent hearing loss of 40 dB or more in both ears. Accordingly, the prevalence of this type of hearing loss in the YHC population was 0.06%.

### 3.2. Interventions

Of the hard of hearing children with bilateral hearing loss who had a loss of 40 dB or more in the best ear, and were born in 2014–2016, 94 were known to the NSDSK early intervention center. It is striking that a substantial percentage of these children had not been detected by the NHS. Table 5 shows the backgrounds of these children.

The average age at which children attended an intake appointment at the early intervention center was 6 months, with a very wide spread of 1.1 to 31 months. At the start of early intervention, the average age of the children was 6.6 months (1.4–32 months). See Table 6. For those children where the date they started early intervention with hearing aids was known, the average age at which they received a hearing aid was 4.5 months. These average values are above the target age of 4 months for starting rehabilitation (hearing aids and/or early intervention).

Where the age of starting an intervention is exceeded, there is often a clear reason for this. Table 7 illustrates the causes of delays in the process. In most cases, the child did not originally appear to belong to the early intervention target group.

These were children in whom unilateral hearing loss was originally found, or in whom hearing loss of a temporary nature was suspected. After some time it became clear that these children required early intervention. Table 6 shows the results if this sub-population is excluded. These are, of course, better because they are children identified through NHS followed by a straight forward diagnostic process and undisputable diagnosis. The average age of the start of early intervention with hearing aids was then 3.6 months (2.9–5.2), the average age of intake was 2.3 months (1.1–4.0), and the average age at the first home visit was 3.1 months (1.4–5.8). Additionally, 98% of children had had the intake before the age of 4 months, and 77% had had the first home visit.

## 4. Discussion

The quality of the delivery of the NHS program is safeguarded at two levels—at screener level by the regional coordinator, and at national organizational level by the RIVM-CvB. If necessary, the regional coordinator gives feedback to the screeners. Each year, the RIVM-CvB sends national monitoring reports to all youth health services organizations and SHCs. A letter setting out the main results and key points for further attention is included in this. The YHC, the SHCs, and the institutes for early intervention each feel responsible for their own link in the NHS chain. They are open to discussing quality control and points for improvement. If the NHS has taught us anything about factors of success for screening programs, it is that continued focus, setting high standards, and working with specialized dedicated teams is crucial.

Long-standing key points for improvement were around timely completion of diagnostic testing and the timely start of an intervention if necessary. After the introduction of the NHS, Leiden University Medical Center’s DECIBEL study reported on the ages at which children first received a hearing aid [11]. Of all the children born in 2005, in regard to the time of the NHS implementation in the Netherlands, 50% of all children detected as having permanent hearing loss had hearing aids before the age of 6 months. The average age of being fitted with a hearing aid was 7.5 months, with a wide range (2–57 months), despite the diagnosis being known at an average age of 2.1 months [11].

In 2008, 2010 and 2012, RIVM-CvB commissioned NSDSK to carry out an evaluation of the timeliness of the NHS program for fitting hearing aids in newborns in whom hearing loss had been detected. Their analyses showed that in 2012, the average age of fitting a hearing aid was 5.3 months, and 55% of all children diagnosed as having permanent hearing loss had hearing aids before the age of 4 months. In comparison with 2005, this was a considerable improvement—however, these analyses were based on relatively incomplete data from the feedback reports of the SHCs about the ages at which hearing aids were fitted. In 2017, the RIVM-CvB commissioned a study from the Platform for Audiological Clinical Testing of the Federation of Dutch Audiology Centers. They investigated the timeliness of hearing aid rehabilitation in children born in 2014. In addition, for the first time, timeliness was included in the early intervention program. An important factor of the study was that an intervention (hearing aids/family counselling) had been started with all children who had a hearing loss of 60 dB or more, at around the age of 4 months. Interventions were given later for children with less severe degrees of hearing loss. This was partially due to the fact that some parents had difficulty accepting the diagnosis. In addition, logistical factors of the SHCs and co-morbidity and/or illness in the children were found to play a role [12]. The results of our current study presented in this article show that in the absence of further problems or conditions, and following the standard route (i.e., NHS, diagnostics and intervention), the age at which first-time interventions were given was 3.2 months, which is well within the targeted timeframe.

However, the high quality of the NHS program in the Netherlands is not a reason to sit back and relax. In practice, continual adaptations are necessary and developments that could potentially influence the quality of the program which arise need to be addressed, as was the case with the introduction of new screening devices that required a new training program and instruction materials. A new European privacy law (General Data Protection Regulation) was introduced in May 2018, thus making it necessary to adjust the program to meet the specific requirements of this law. One important point for the YHC is that the NHS does not guarantee lifelong good hearing. The OAE screening cannot detect hearing loss if the cause lies within the brain, which does occur occasionally. In addition, there are some cases where the hearing loss manifests itself after the screening has taken place, or where initially minor hearing loss progressively worsens over time. Thus, it is important that the YHC, parents, and others involved in the care of the child remain aware of the possibility of hearing loss developing after the neonatal period. The Dutch YHC guidelines on the early detection of hearing loss in children and young people (0–18 years) offers more guidance on this matter.

## 5. Conclusions

The NHS program, carried out by the YHC, is a long-standing, stable screening program with a demonstrably high level of quality. Results have shown that participation levels were high, while refer percentages stayed low. Only 0.3% of screened children were referred for further testing, and 35–40% of them were proven to have unilateral or bilateral hearing loss of a minimum of 40 dB. Where timeliness is concerned, there is still room for improvement in both diagnostics and the start of intervention. The average age of children starting out in the early intervention program was 6.6 months (1.4–32 months). For children whose early intervention starting date with hearing aids was known, the average age at which they got a hearing aid was 4.5 months, and these averages are above the target of 4 months for starting rehabilitation.

## Figures and Tables

**Table 1:** Participation in screening and diagnostics 2002/2006–2016.

	Norm	2002–2006	2008	2009	2010	2011	2012	2013	2014	2015	2016
1st screening (OAE)	≥95%	98.6%	99.3%	99.3%	99.3%	99.4%	99.4%	99.4%	99.5%	99.6%	99.7%
2nd screening (OAE)	≥97%	98.8%	99.4%	99.5%	99.4%	99.3%	99.3%	99.4%	99.3%	99.5%	99.7%
3rd screening (AABR)	≥98%	99.0%	99.3%	99.6%	99.3%	98.8%	99.7%	99.6%	99.9%	99.8%	99.7%
Diagnostics	100%	**93.1%**	**95.2%**	**93.9%**	**93.4%**	**95.3%**	**94.9%**	**96.5%**	**95.7%**	**96.6%**	**95.4%**

Values in bold do not meet the target values. N.B. 2002–2006 data originate from nation-wide implementation ([2] Data on other years originate from the national monitors on neonatal hearing screening from The Netherlands Organization for Applied Scientific Research (TNO) commissioned by The Dutch Institute for Public Health and the Environment’s Center for Population Screening (RIVM-CvB) [7].

**Table 2:** Referrals per screening and for diagnostics 2002/2006–2016.

	Norm	2002–2006	2008	2009	2010	2011	2012	2013	2014	2015	2016
1st screening (OAE)	≤7%	6.3%	5.1%	4.7%	4.6%	4.6%	4.5%	4.4%	4.3%	4.3%	4.6%
2nd screening (OAE)	≤40%	36.4%	35.2%	35.9%	36.5%	36.2%	35.7%	34.4%	33.1%	34.1%	33.2%
3rd screening (AABR)		12.5%	16.5%	16.9%	16.2%	17.9%	18.1%	19.1%	18.5%	18.3%	18.6%
Diagnostics	≤0.5%	0.29%	0.30%	0.29%	0.27%	0.30%	0.29%	0.29%	0.26%	0.27%	0.29%

N.B. 2002–2006 data originate from nation-wide implementation [2]. Data on other years originate from the national monitors on neonatal hearing screening from TNO commissioned by the RIVM-CvB [7].

**Table 3:** Timeliness of screening and diagnostics 2002/2006–2016.

	Norm	2002–2006	2008	2009	2010	2011	2012	2013	2014	2015	2016
1st screening (OAE)	<28 days >97%	**95.6%**	98.0%	98.2%	98.2%	98.4%	98.5%	99.1%	99.2%	99.4%	99.4%
2nd screening (OAE)	<35 days >95%	-	**93.7%**	**94.4%**	**93.7%**	**94.3%**	**94.6%**	97.0%	97.7%	97.9%	98.0%
3rd screening (AABR)	<42 days ≥95%	**81.2%**	**90.4%**	**91.5%**	**90.4%**	**91.1%**	**91.9%**	95.7%	97.0%	96.6%	97.0%
Diagnostics	<92 days >95%	**80.3%**	**84.6%**	**77.2%**	**74.8%**	**78.8%**	**84.8%**	**84.3%**	**83.4%**	**85.9%**	**85.1%**

Values in bold do not meet the target value. N.B. 2002–2006 data originate from nation-wide implementation [2]. Data on other years originate from the national monitors on neonatal hearing screening from TNO commissioned by the RIVM-CvB [7].

**Table 4:** Number of diagnoses of unilateral and bilateral hearing loss 2002/2006–2016.

Diagnosis	2002–2006	2008	2009	2010	2011	2012	2013	2014	2015	2016
Unilateral hearing loss	89	96	82	76	88	91	87	95	82	68
Bilateral hearing loss	128	136	163	115	99	119	113	121	113	128

N.B. 2002–2006 data originate from nation-wide implementation [2]. Data on other years originate from the national monitors on neonatal hearing screening from TNO commissioned by the RIVM-CvB.

**Table 5:** Children not detected by neonatal hearing screening (NHS) under early intervention.

Cause	Number
Neonatal intensive care units (NICU)	14
Child referred by physician/pediatrician shortly after birth	6
Children fully screened by NHS who later became deaf	5
*Hearing loss due to bacterial meningitis infection*	*3*
*Hearing loss due to ototoxic medication*	*2*
Unscreened (in the Netherlands)	3
Screening refused/unscreened due to circumstances	3
**Total**	**31**

**Table 6:** Data of the timeliness of early intervention with hearing aids, obtained from the Dutch Foundation for the Deaf and Hard of Hearing Child (NSDSK) 2014–2016.

**Number of children under early intervention born 2014–2016**			**94**
Number of children detected via NHS YHC			63
Severity of hearing loss			
*Moderate hearing loss (40–60 dB)*			31
*Severe hearing loss (60–80 dB)*			11
*Profound hearing loss (80 dB)*			21
**Age at diagnosis and start of intervention in all children in months**			
Average age at diagnosis	*N* = 63	1.4 (0.3–7.0)	
Average age hearing aids	*N* = 20	4.5 (2.9–12.3)	
Average age at intake for early intervention	*N* = 59	5.9 (1.4–30.9)	
Average age first home visit for early intervention	*N* = 60	6.6 (1.4–32.0)	
**Age in months of diagnosis and start of intervention NHS/early intervention target groups only**			
Average age at diagnosis	*N* = 47	1.1 (0.3–2.8)	
Average age hearing aids	*N* = 15	4.0 (2.9–5.2)	
Average age at intake for early intervention	*N* = 43	2.3 (1.4–4.0)	
Average age first home visit for early intervention	*N* = 44	3.2 (1.4–5.8)	
Percentage hearing aids within 4 months	*N* = 15	73%	
Percentage intake within 4 months	*N* = 43	98%	
Percentage starting early intervention within 4 months	*N* = 44	77%	

**Table 7:** Causes of late intake.

Cause	Number
Child not initially in target group	10
Parents initially refused care	4
Initially at another institution/other care elsewhere	2
**Total**	16
